# Optimization of Patient Management During the COVID-19 Pandemic: Chest CT Scan and PCR as Gatekeepers of the Radiation Therapy Workflow

**DOI:** 10.3389/fonc.2020.556334

**Published:** 2020-11-18

**Authors:** Roger Sun, Samy Ammari, Sophie Bockel, Samir Achkar, Mansouria Merad, Laurent Dercle, Sofia Rivera, Cyrus Chargari, Eric Deutsch

**Affiliations:** ^1^ Gustave Roussy, Département de Radiothérapie, INSERM 1030, Université Paris-Saclay, Villejuif, France; ^2^ Gustave Roussy, Département d’Imagerie Médicale, Université Paris-Saclay, Villejuif, France; ^3^ Gustave Roussy, Département d’Oncologie Médicale, Université Paris-Saclay, Villejuif, France; ^4^ Department of Radiology, Columbia University Irving Medical Center, New York, NY, United States

**Keywords:** coronavirus disease, radiotherapy, chest CT, workflow, PCR

## Introduction

The coronavirus disease 2019 (COVID-19) has been rapidly spreading since the first patients were described in Wuhan, China in late December 2019 (1). As of mid-July 2020, nearly 15 million of confirmed cases and 600,000 deaths have been reported worldwide (2). This global pandemic and international health crisis has challenged healthcare providers to profoundly re-organize healthcare systems in order to ensure the continuity of essential treatments, while limiting the risk to patients and healthcare providers, and simultaneously handling shortages in personnel, beds, and equipment (3).

Increasing evidence suggests that cancer patients and especially those undergoing treatment might be at higher risk of developing severe forms of COVID-19 (4–7). Indeed, anticancer treatment such as chemotherapy and radiotherapy that induce neutropenia and/or lymphopenia, as well as targeted therapy or immune-check point inhibitors, may worsen the course of COVID-19, although this is still debated (3, 8) For this reason, cancer societies have recommended to adapt the management of cancer patients by de-escalating cytotoxic chemotherapy, delaying non-urgent treatments, and considering non-surgical options when feasible (9, 10). In radiation oncology, some indications remain non-deferrable, such as chemoradiotherapy for locally advanced tumors, brachytherapy, and urgent palliative treatment, particularly in patients with rapidly growing tumors (11). In addition, radiotherapy capacity may even need to be increased as an alternative treatment to compensate for surgery cancellations. While hypofractionated radiotherapy schedules may help to limit the number of hospital visits, daily sessions for at least 2 weeks remain the rule for most curative treatments, with some plans lasting up to 7–8 weeks (12, 13). Moreover, strategy relying only on patient selection and prioritizing treatment management alone may not be sufficient in a prolonged healthcare crises (10). Active and effective strategies of early detection of COVID-19 should therefore be discussed and tailored for radiation oncology departments, especially since a significant proportion of patients are asymptomatic (14).

## SARS-CoV-2 Detection and COVID-19 Diagnosis

COVID-19 is an infectious respiratory disease caused by a novel coronavirus called SARS-CoV-2 (15). The accepted standard routine laboratory method to diagnose COVID-19 consists of the use of reverse transcription polymerase chain reaction (rt-PCR) to detect viral RNA in respiratory samples. However, performance of nasopharyngeal and oropharyngeal swabs is questioned since several studies have shown a high number of false negative results (16, 17). In a cohort of 213 patients with mild to severe symptoms of COVID-19, Yang et al. reported positive rates ranging from 73.3% for nasal swabs performed less than 7 days after illness onset on patients with severe symptoms and 53.6% for nasal swabs performed more than 8 days after illness onset on patients with mild symptoms. Wang et al. reported low positive rates of 32% for pharyngeal swabs (126/398 patients) and 63% for nasal swabs (5/8 patients). Moreover, rt-PCR has other limitations including the availability of testing kits, the need for adapted infrastructure, training of personnel for high quality swab samples, and relative lengthy turnaround times for test results.

On the other hand, some authors have suggested computed tomography (CT) could be pivotal for the diagnosis and the screening of COVID-19 (18, 19). In a cohort of 1,014 patients, Ai et al. reported a positive rate of 59% for rt-PCR (601/1014) and 88% for chest CT (888/1014), with 97% of positive rt-PCR patients having also a positive chest CT (580/601) (18). The higher sensitivity of chest CT compared to rt-PCR was confirmed in a recent meta-analysis [94% (5% CI: 91%, 96%) vs. 89% (95% CI: 81%, 94%), respectively] (20). However, this study highlighted the low specificity of chest CT [37% (95% CI: 26%, 50%)] with poor positive predictive value (PPV) for populations with low expected prevalence of COVID-19, limiting its routine use for mass screening and diagnosis in the overall population, especially since CT scans also expose people to ionizing radiation. Therefore, the general consensus is to not recommend chest CT for diagnosing COVID-19 and to reserve CT for patients with worsening symptoms (21, 22). However, several authors have suggested that imaging could help patient triage in a resource-constrained environment, especially when the pre-test probability of COVID-19 is high (21, 22). Nevertheless, with a high negative predictive value (NPV) estimated between 90.6% and 99.8%, chest CT imaging could still be very valuable in specific situations, such as in screening patients undergoing radiotherapy.

Recently, serological tests have become more widespread (23). Detection of IgM antibodies associated with symptoms is highly suggestive of SARS-CoV-2 infection. IgM antibodies may be detectable around 5 to 10 days after the onset of symptoms, with a mean time for seroconversion of 10 to 12 days for IgM, and 12 to 14 days for IgG (23–26). However, whether an antibody response with neutralizing antibodies is associated with protective immunity is still unclear, and higher IgG concentration have been reported in patients with severe COVID-19 infection (27). While these tests were not recommended for systematic screening by the World Health Organization (28), they were recommended by the French Haute Autorité de Santé in patients with negative RT-PCR and clinical suspicion for COVID-19 (29).

## Optimizing Medical Imaging in Radiotherapy During the COVID-19 Pandemic

### Feasibility and Benefit of Chest CT in Radiation Therapy

Radiation therapy requires each patient to undergo a simulation CT scan for treatment planning. This CT allows the visualization and delineation of target tumor volumes to irradiate and normal tissues to spare, the calculation and the optimization of the radiation dose, and the reproducibility of patient positioning (30). An additional chest CT scan or extending the scan coverage of a simulation CT could therefore be easily performed for each patient without modifying patient’s visit or the radiotherapy workflow. Moreover, the expected additional delivered radiation dose to the chest is low, around 12 mGy [interquartile range (IQR), 7–17 mGy] (31, 32), and may be considered as negligible for patients undergoing radiotherapy. Indeed, even for non-thoracic treatment, the estimated dose at 30 cm from the irradiated field is estimated to be 0.05%–0.7% of the delivered dose (33). For example, the estimated dose received to the chest for a pelvic treatment of 45 Gy would correspond to 2–26 times the dose of a chest CT. In addition, free-breathing chest CTs are routinely performed during the CT simulation for patients with lung and breast cancers, metastases to the thoracic and lumbar spine, and sometimes for head and neck cancers. In certain indications, such as in breast cancer, deep inspiration breath hold radiotherapy technique allows acquisition of breath-hold chest CT.

For patients with other cancers, the additional chest CT could also serve as a baseline for reference, in addition to screening for COVID-19. Indeed, cancer patients may have progressive cancer- or cancer-treatment related lung abnormalities, which may be complicated to interpret if the patient becomes symptomatic. Moreover, the rate of incidental findings in cancer patients may be relatively high: a study of 510 patients reported incidental findings in up to 28% of patients, including 3.4% of patients with a significant finding that either changed the cancer therapy or required immediate treatment (34).

### Early Detection of COVID-19 in Patients Undergoing Radiotherapy

The lack of large epidemiological studies and the difficulty of estimating the true incidence of COVID-19 cases make it challenging to determine whether cancer patients are more or less vulnerable to SARS-CoV-2 in comparison with the overall population. Indeed, among COVID-19 patients, an important proportion of patients are asymptomatic, but may still be contagious (35–37). A voluntary screening on 10,797 patients in Iceland, with 87 COVID-19 positive patients (0.8%) of whom 57% were asymptomatic (14). Moreover, with an incubation period between 3 to 6 days (15, 38), a study estimated that up to 44% [95% CI (25–69)] of secondary cases may have been infected by pre-symptomatic cases (36). Cancer patients undergoing treatment may have compromised immunity, potentially increasing their morbidity and mortality from COVID-19. Miyashita et al. reported 5,688 COVID-19 patients, including 334 patients with cancer. Intubation was more frequent in cancer patients [RR: 1.89 95% CI (1.37–2.61)], but there was no significant excess risk of death (5). Kuderer et al. reported in a study of 928 patients with history of cancer that there was an increased 30-day mortality in patients with active cancer [progressing vs. remission: Odds ratio = 5.20, 95% CI (2.77–9.77)] (39).

Radiotherapy treatment requires frequent hospital visits for the patients, a flow of multiple patients per day on the same machine, and involvement of an entire team of healthcare professionals including a medical dosimetrist, medical physicist, radiation oncologist, radiation therapists, oncology nurses, caregivers, and medical secretaries. Strategies for early detection of COVID-19 are therefore of utmost importance to ensure patient and medical staff safety during the COVID-19 pandemic, especially in regions with high prevalence and documented community spread.

### Proposal of COVID-19 Testing for Asymptomatic Patients in Radiation Oncology Departments

**Figure 1:**
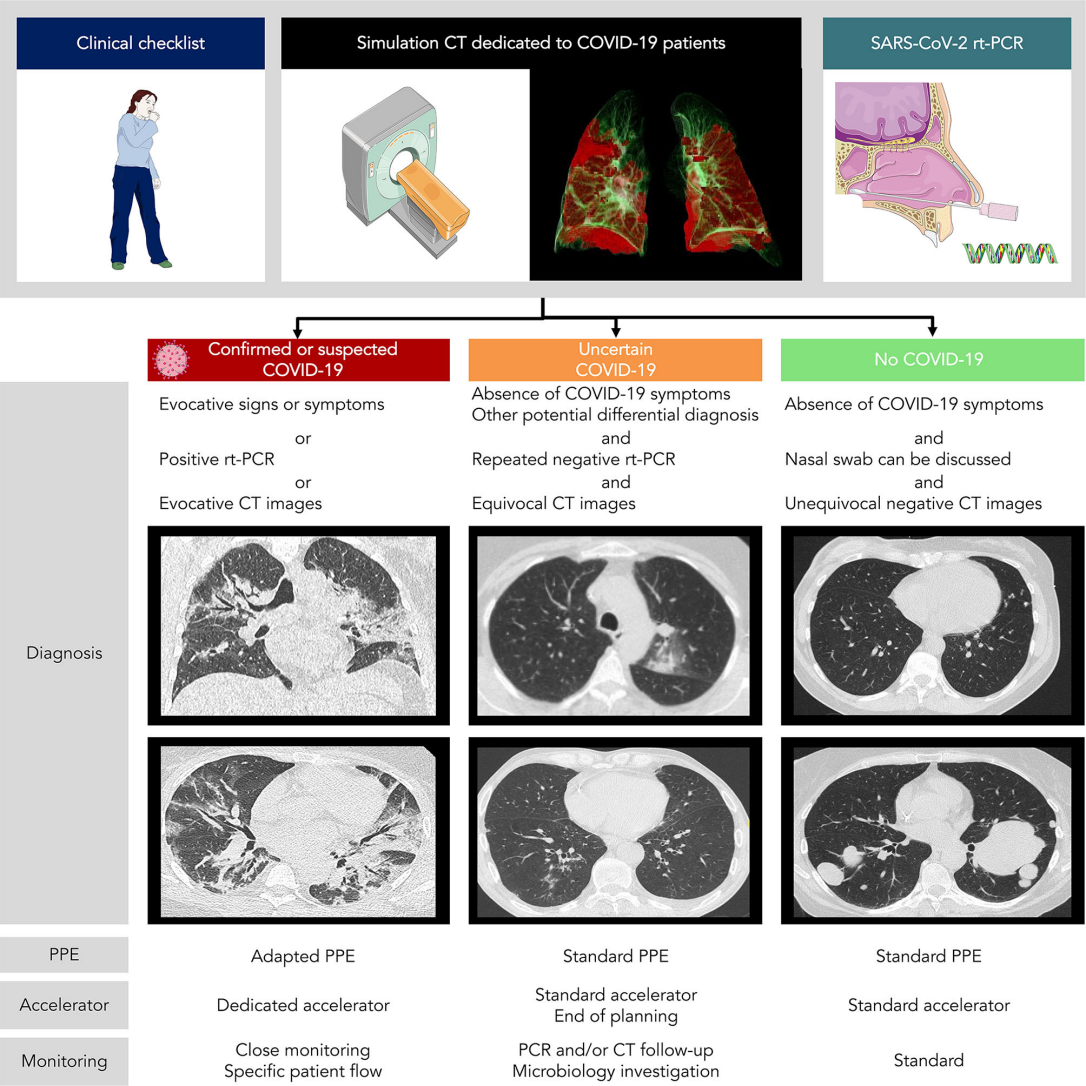
COVID-19 patients management in radiotherapy according to chest CT + rt-PCR screening. PPE, personal protective equipment.

A workflow including systematic nasopharyngeal swab and chest CT for asymptomatic patients is currently being evaluated in our Radiotherapy department, as the prevalence of COVID-19 was high in our region, with up to 11.9% of individuals infected (range: 7.6% to 19.4%) according to epidemiologic models (40). Inspiratory breath hold chest acquisition is made during the simulation CT, with the same CT acquisition protocols conventionally used in radiology. While free-breathing acquisitions are usually done in radiotherapy, they are more susceptible to motion artifacts and may obscure or even mimic subtle ground-glass opacities commonly seen in COVID-19, and regular cone-beam CT (CBCT) positioning review may require comparison with reference chest CT. Images are then reviewed by both the treating radiation oncologist and an experienced radiologist for imaging findings suggestive of COVID-19 such as ground-glass opacity, condensation, reticulation, interlobular or intralobular septal thickening, nodules, and distribution of the lesions (41). For suspected COVID-19 patients on either radiological findings or clinical symptoms but negative RT-PCR, directed nasopharyngeal swab and inflammatory blood test are repeated before the onset of radiotherapy. This procedure allows the classification of patients into three different categories with specific management according to the COVID-19 probability (**Figure 1**):

1) Confirmed or probable cases: We propose using a dedicated treatment room with adapted personal protective equipment {protective masks against inhalation of droplets, such as FFP2 (filtering facepiece type 2) masks [standard NF EN 149 (42)], gown, gloves, eye protection, and apron} and a dedicated accelerator for the irradiation of suspected and/or confirmed COVID-19 patients, which may help to avoid deferring treatment. On a case by case basis taking into account symptoms, deferring radiotherapy after the acute phase of infection is discussed. However, this approach using a dedicated room for confirmed or probable cases is only feasible in large centers with a high number of accelerators.
2) Uncertain cases: We propose in these patients close monitoring of clinical symptoms and repeating nasopharyngeal swabs. Further investigation might be proposed according to the multidisciplinary team, such as CT follow-up or other microbiology investigation and specimens. These patients are preferably scheduled for treatment at the end of the day.
3) Unlikely cases: We propose using standard personal protective equipment and routine radiotherapy treatment protocol.

In addition to CT scan and rt-PCR screening, we have undertaken a global reorganization of our radiation oncology department with implementation of barrier precautions and social distancing. Prior to entry, patients are asked to answer a short checklist to screen for clinical symptoms, such as fevers, chills, cough, etc., and reception staff measure the temperature of patients at the entrance. All medical staff and patients are required to wear surgical masks in the department. The flow of patients in the radiation oncology department has also been rethought. The simulation CT is dedicated to COVID-19 patients only on Friday, allowing a two-day interval before using the scanner again for patients without COVID-19. Whenever possible, we encourage COVID-19 patients to wait for their session in their car or in the taxi in order to limit the time spent in the waiting room, and therefore, limiting the risk of SARS-CoV-2 transmission.

These screening strategies have the most potential and maximum benefit during the peak of COVID-19 cases and adjustments need to be made according to the evolution of the pandemic and the pre-test probability of COVID-19 in a particular area, as low prevalence of COVID-19 leads to a low PPV, especially for chest CT (20). For instance, while systematic baseline RT-PCR testing is recommended in Canada by the Ontario provincial Ministry of Health guidelines for cancer patients undergoing immunosuppressive cancer treatment, including radiation therapy, high priority testing criteria for asymptomatic patients in the event of testing limitations were defined, included age ≥ 60 years, performance status ≥ 2, comorbid conditions or impaired immunity, significant smoking history and lung tissue in the radiation treatment volume (43).

## Conclusion

The COVID-19 pandemic has significantly impacted the delivery of care to cancer patients, leading to delays in providing adequate treatment. While postponing treatments and providing remote consultations were feasible in the early stages of the pandemic, these temporary measures are no longer sustainable as the pandemic continues. Hospitals and medical services need to develop and adopt long-term strategies to continue providing cancer care during the pandemic. In patients undergoing radiotherapy, we propose using chest CT and rt-PCR screening for early detection of COVID-19, especially since a number of patients are asymptomatic and cancer patients might be more vulnerable than the overall population. Rethinking the flow of patients is also critical to allow the continuation of care, with implementation of barrier precautions and social distancing.

## Author Contributions

RS and ED prepared the first draft of the manuscript. LD conceived the figure. SB, SR, SAm, MM, SAc, LD, and CC reviewed the manuscript. All authors contributed to the article and approved the submitted version.

## Conflict of Interest

The authors declare that the research was conducted in the absence of any commercial or financial relationships that could be construed as a potential conflict of interest.

The handling editor is currently organizing a Research Topic with the authors SA and LD.
